# Improving Detection of Cognitive Decline and Impairment Using Everyday Behavioral and Social Markers

**DOI:** 10.1093/geroni/igaa057.2012

**Published:** 2020-12-16

**Authors:** Ruixue Zhaoyang, Eric Cerino, Stacey Scott

## Abstract

Early detection of cognitive decline and mild cognitive impairment (MCI) during the pre-symptomatic phase of Alzheimer’s disease is particularly important for maximizing effectiveness of clinical trials and efficiency of resource allocation. However, it is difficult to distinguish early signs of decline and impairment from normative aging, especially with biomarkers and clinical-based assessments that are expensive and challenging to apply widely. Ambulatory assessments in naturalistic settings provide opportunities to capture everyday markers of cognitive decline and offer cost-effective tools for sensitive, early detection of transitions to MCI in community-dwelling older adults. In this symposium, we present four studies that use ecological momentary assessment (EMA) data from the Einstein Aging Study to showcase how everyday markers of behavioral and social functioning assessed up to six times a day for 14 consecutive days can facilitate early detection of cognitive difficulties. Zhaoyang et al. examine whether older adults with intact cognition versus MCI differ in patterns of daily social interactions. Hyun et al. investigate how the diversity of daily activities is associated with ambulatory cognitive deficits. Cerino et al. compare the sensitivity of everyday markers of stress versus global trait-based stress measures to detect MCI. Roque et al. use completion time from EMA surveys as a reliable and unobtrusive way to measure cognition and distinguish those with and without MCI. Stacey Scott will integrate insights gained from these four papers, and discuss the opportunities and challenges faced when combining ambulatory assessments of everyday markers with traditional methods to better detect cognitive decline and impairment.

